# Data showing chemical compositions of the essential oils of the leaves of *Cymbopogon citratus* obtained by varying pH of the extraction medium

**DOI:** 10.1016/j.dib.2016.06.019

**Published:** 2016-06-22

**Authors:** E.O. Ajayi, A.P. Sadimenko, A.J. Afolayan

**Affiliations:** a Department of Chemistry, University of Fort Hare, Alice 5700, South Africa; b MPED Research Center, Department of Botany, University of Fort Hare, Alice 5700, South Africa

**Keywords:** Essential oil, *Cymbopogon citratus*, pH Extraction medium

## Abstract

This article describes the various chemical components as obtained from the oils in the leaves of *Cymbopogon citratus* using hydrodistillation and solvent-free microwave extraction methods. Furthermore, extractions of the oils were also carried out with a slight in pH variation and compared, “GC–MS evaluation of *C. citratus* (DC) Stapf oil obtained using modified hydrodistillation and microwave extraction methods” (Ajayi et al., 2016 [1]). The current article contains one table exhibiting a list of compounds in the four different methods of extraction. Comparative studies amongst the various methods of extraction are highlighted in the table.

**Specifications Table**

**Table:** 

Subject area	Chemistry
More specific subject area	Essential oil extraction and modified hydrodistillation
Type of data	Table
How data was acquired	Experiments performed on Agilent 6890 GC, coupled to an Agilent 5975 MSD
Data format	Raw, analyzed
Experimental factors	Plant matrixes were thoroughly washed prior extraction.
Experimental features	For pH variation in modified hydrodistillation, 5% solution each of citric acid and sodium bicarbonate was used to effect oil extraction from the plant.
Data source location	Nkonkobe Local Municipality, Eastern Cape, University of Fort Hare
Data accessibility	Data are present with this article.

**Value of the data**

• The data highlight and point out various chemical compounds obtained in acid and base media, which are absent in water-distilled medium. The data are remarkable, indicating that pH variation brings about the extraction of important compounds which are absent in both water-distilled and microwave extraction.
• pH variations in essential oil extraction is certain to influence the various chemical components when compared with the conventional method of hydrodistillation.
• The data present more components in the pH media than water distilled and microwave extraction.

## 1 Data

The data presented here is a Table showing the various chemical compounds obtained from different media of extraction.

## 2 Experimental design, materials and methods

The data were obtained by carefully distilling the plant materials using different extraction media which are water-distilled, microwave extraction, acid-distilled and alkaline-distilled. Slight variation of acid and base (5% each of citric acid and sodium bicarbonate). Most of the compounds exist in trace amount with exception of the high molecular alkanes, obtained from the microwave extraction method.

The various chemical components were analyzed and identified by GC–MS analysis as previously described [1]. Data was gathered with Chem station. (Table 1).

## Figures and Tables

**Table 1:** Chemical components of *Cymbopogon citratus* using modified hydrodistillation (pH variation) and solvent-free microwave extraction. (RT & %A are the Retention Times and % Peak Areas respectively).

**No.**	**Name of compound**	**Molecular formular**	**Molecular weight**	**Oil extraction method**							
				**Water**		**Microwave**		**Acidic**		**Basic**	
				**RT**	**%A**	**RT**	**%A**	**RT**	**%A**	**RT**	**%A**
1	β-Ocimen	C_10_H_16_	136	4.38	0.48	–	–	–	–	–	–
2	Vinyl cyclohexane	C_8_H_14_	110	5.81	1.78	–	–	–	–	–	–
3	ε-Cyclogeraniolene	C_9_H_16_	124	5.60	0.73	–	–	–	–	–	–
4	Geraniol acetate	C_12_H_20_O_2_	196	8.14	0.33	–	–	–	–	8.14	0.21
5	Caryophyllene	C_15_H_25_	204	8.88	0.48	–	–	8.89	0.29	–	–
6	Selina-6-en-4-ol	C_15_H_26_O	222	11.03	0.36	–	–	11.04	0.24	11.03	0.41
7	Neryl propanoate	C_13_H_22_O_2_	210	13.16	0.77	–	–	–	–	–	–
8	4-Carene	C_10_H_16_	136	–	–	–	–	4.38	1.31	–	–
9	Lavandulol	C_10_H_18_O	154	–	–	–		6.71	1.35	–	–
10	trans-2-Caren-4-ol	C_10_H_16_O	152	–	–	–	–	–	−	7.20	1.82
11	1-octanone, -(2-furanyl)-	C_12_H_18_O	194	–	–	8.10	1.66	–	–	–	–
12	N-(*p*-Methoxyphenyl)maleimide	C_11_H_9_NO_3_	203	14.12	1.59	–	–	–	–	–	–
13	(1S, 4R)-p-Mentha-2, 8-diene,	C_10_H_16_O_2_	168	13.85	0.80	–	–	–	–	–	–
	1-hydroperoxide										
14	2H-Pyran, tetrahydro-4-methyl-	C_10_H_18_O	154	–	–	14.34	1.06	–	–	–	–
	2-(2-methyl-1-propenyl)-										
15	2-Hexanoic acid, 3, 4, 4-trimethyl-	C_9_H_14_O_3_	170	–	–	8.10	1.66	–	–	–	–
	5-oxo, (Z)-										
16	2, 6-Dimethyl-1, 3, 5, 7-	C_10_H_14_	134	–	–	–	−	4.06	0.65	–	–
	octatetraene, E,E-										
17	1, 2, 3, 1’, 2’, 3’-Hexamethyl-	C_16_H_26_	218	–	–	–	–	–	−	5.60	0.63
	bicyclopentyl-2, 2’-diene										
18	Squalene	C_30_H_50_	411	–	–	–	–	–	−	14.02	0.47
19	2H-Pyran-2-carboxylic acid, 5-	C_10_H_12_O_4_	196	15.36	0.85	–	–	–	–	–	–
	ethylidene-5, 6-dihydro-2, 3-										
	dimethyl-6-oxo, [S-(E)]-										
20	1, 7-Octadien-3-one, 2-methyl-6-	C_10_H_14_O	150	–	−	14.78	1.59	–	–	–	–
	methylene.										
21	3, 7, 11-tridecatrienoic acid, 4,	C_17_H_28_O_2_	264	–	–	–	–	–	−	14.02	0.47
	8, 12-trimethyl-, methyl ester, (Z,E)-										
22	2-(1-Cyano-2-methyl-propylamino)	C_10_H_17_N_3_	179	–	−	14.78	1.59	–	–	–	–
	−3-methyl-butyronitrile										
23	n-Hexadecanoic acid	C_16_H_32_O_2_	256	–	–	–	−	13.25	0.80	–	–
24	11, 14, 17-Eicosatrienoic acid	C_21_H_36_O_2_	321	–	–	–	−	14.23	0.79	–	–
25	1-Propanone, 2-chloro-1-	C_12_H_15_ClO	211	–	–	–	−	13.73	0.96	–	–
	(2, 5-dimethylphenyl)−2-methyl-										
26	Octacosane	C_28_H_58_	395	–	−	14.51	4.51	–	–	–	–
27	Heneicosane	C_21_H_44_	297	–	−	14.51	4.51	–	–	–	–
28	5-Hepten-2-one, 6-methyl-	C_8_H_14_O	126	–	–	–	–	–	−	3.78	0.44
29	Oxalic acid, 2-methylphenyl	C_24_H_38_O_4_	391	–	–	–	–	–	−	3.78	0.44
	pentadecyl ester										
30	Phenol, 2, 3, 5, 6-tetramethyl	C_10_H_14_O	150	–	–	–	–	–	−	4.83	0.14
	(Durenol)										
31	Histamine	C_5_H_9_N_3_	111	5.50	0.34	–	–	–	–	–	–
32	2, 4-Heptadienal, (E,E)-	C_7_H_10_O	110	–	–	–	–	–	−	5.81	0.50
33	p-Menth-1(7)-en-9-ol	C_10_H_18_O	154	13.70	0.50	–	–	–	–	–	–
34	Acetic acid, 1, 3, 7-trimethylocta-	C_13_H_22_O_2_	210	14.49	0.24	–	–	–	–	–	–
	2,6 dienyl ester										
35	Farnesol	C_15_H_26_O	222	14.65	0.30	–	–	–	–	–	–
36	Tricyclo[7.2.2.0(3, 8]−5, 12-dien-	C_17_H_22_O_4_	290	15.81	0.41	–	–	–	–	–	–
	2-one, 1-methoxy-4-(acetoxy) methyl-										
37	5-(4-trifluormethylsulfonylphenyl	**C**_**17**_**H**_**19**_**F**_**3**_**N**_**2**_**O**_**2**_**S**	372	–	–	–	–	–	−	13.76	0.12
	azo)−1, 2, 3, 4, 5-pentamethylcyclopentadiene										
38	Cobalt, (.eta.4-8-ethylidene-2, 6-	C_20_H_27_CoSe^−2^	405	–	–	–	–	–	−	13.91	0.05
	Cyclooctadiene-1-selone-Se) [1, 2,										
	3, 4, 5-.eta.)−1, 2, 3, 4, 5-pentamethyl-2, 4-cyclopentadien-1-yl]-										
39	1-(beta.-d-Ribofuranosyl)-4-difluoromethyl-5-bromouracil	**C**_**10**_**H**_**11**_**BrF**_**2**_**N**_**2**_**O**_**6**_	373	–	–	–	–	–	−	13.91	0.05
40	Di(n-pentyloxy)methylsilane	C_14_H_16_	184	–	–	–	–	–	−	13.97	0.01
41	2,6, 10-Undecatrien-8-ol, 2, 6-dimethyl	C_13_H_22_O	194	–	–	–	–	–	−	14.75	0.13
42	cis, trans-4-n-propyl-3-oxabicyclo	C_12_H_22_O	182	–	–	–	−	3.73	0.43	–	–
	[4.4.0]decane										
43	1, 2, 4, 4-Tetramethylcyclopentene	C_9_H_16_	124	–	–	–	−	3.94	0.38	–	–
44	Ethanone, 1-(1, 4-dimethyl-3-cyclo	C_10_H_16_O	152	–	–	–	−	4.20	0.31	–	–
	hexen-1-yl)-										
45	Linalool oxide	C_10_H_18_O	170	–	–	–	−	4.69	0.11	–	–
46	Cyclopentanepropanol, 2-methylene-	C_9_H_16_O	140	–	–	–	−	4.73	0.17	–	–
47	Bicyclo[3.1.0]hexan-3-ol, 4-methyl-	C_10_H_18_O	154	–	–	–	−	5.55	0.65	–	–
	1-(1-methylethyl)-, (1α, 2β, 4β, 5α)-										
48	Spiro[4.5]decan-2-one	C_10_H_16_O	152	–	–	–	−	5.60	0.27	–	–
49	Methanone, (4-methoxyphenyl) (1,	C_16_H_20_O_2_	244	–	–	–	−	5.68	0.67	–	–
	2, 3-trimethyl-2-cyclopentenyl)										
50	2-Cyclohexen-1-ol, 2-methyl-5- (1-	C_10_H_16_O	152	–	–	–	−	5.92	0.37	–	–
	methyethenyl)-, cis-										
51	cis-Carveol	C_10_H_16_0	153	–	–	–	−	6.26	0.41	–	–
52	3-Cyclohexene-1-acetaldehyde, α,	C_10_H_16_O	152	–	–	–	−	7.27	0.51	–	–
	4-dimethyl-										
53	Ethanone, 1-(2-furanyl)-	C_6_H_6_O_2_	110	–	–	–	−	7.43	0.31	–	–
54	Fenchone	C_10_H_16_O	152	–	–	–	−	8.37	0.30	–	–
55	5-Octen-2-yn-4-ol	C_8_H_12_O	124	–	–	–	−	8.52	0.17	–	–
56	Farnesene	C_15_H_24_	204	–	–	–	−	8.94	0.30	–	–
57	b-Cadinene	C_15_H_24_	204	–	–	–	−	9.63	0.40	–	–
58	Cyclohexadecane	C_16_H_32_	224	–	–	–	−	12.78	0.18	–	–
59	Benzene, 1, 1’-(1, 1, 2, 2-tetra-	C_20_H_26_	266	–	–	–	−	12.85	0.13	–	–
	methyl-1, 2-ethanediyl)bis[4-methyl-										
60	1-Pyrazinyl-4-methyl-2-pentanol	C_10_H_16_N_2_O	180	–	–	–	−	12.90	0.23	–	–
61	trans-β-Santolol	C_15_H_24_O	220	–	–	–	−	12.90	0.23	–	–
62	N-Chlorosuccinimide	C_4_H_4_ClNO_2_	134	–	–	–	−	13.04	0.43	–	–
63	Phenol, 2-(4-diethylaminophenyl-	C_17_H_20_N_2_O	268	–	–	–	−	13.30	0.80	–	–
	iminomethyl)-										
64	1H-Benzimidazol-2-amine	C_7_H_7_N_3_	133	–	–	–	−	13.55	0.13	–	–
65	Silane, diethoxydimethyl-	C_6_H_16_O_2_Si	148	–	–	–	−	13.64	0.12	–	–
66	Benzenepropanol, 2,4,6-trimethyl-	C_12_H_18_O	178	–	–	–	−	13.80	0.11	–	–
67	2-Carene	C_10_H_16_	136	–	–	–	−	4.85	0.77	–	–
68	2-(Trifluoromethyl)benzothiazole	C_8_H_4_F_3_NS	203	–	–	–	−	14.12	0.09	–	–
69	Durene	C_10_H_14_	134	–	–	–	−	14.16	0.14	–	–
