# Supplementary material for: Data showing chemical compositions of the essential oils of the leaves of Cymbopogon citratus obtained by varying pH of the extraction medium
Source: Data Brief. 2016 Jun 22;8:599–604. doi: 10.1016/j.dib.2016.06.019 (PMC4936328; doi:10.1016/j.dib.2016.06.019)
Supplement: Supplementary file 1 — Transparency document [file mmc1.docx]

Dear Editor,

The authors (EO Ajayi, AP Sadimenko, and AJ Afolayan) for the manuscript titled “ Data showing chemical compositions of the essential oils of the leaves of *Cymbopogon citratus* obtained by varying pH of the extraction medium”, reference **DIB-D-16-00310R1** hereby declared that they have no Conflict of Interest regarding the manuscript.

Thank you.
